# Sex chromosome trisomies are not associated with atypical lateralization for language

**DOI:** 10.1111/dmcn.13929

**Published:** 2018-06-10

**Authors:** Alexander C Wilson, Dorothy V M Bishop

**Affiliations:** ^1^ Department of Experimental Psychology Oxford University Oxford UK

## Abstract

**Aim:**

To test the hypothesis that sex chromosome trisomies (SCTs) are associated with reduced left lateralization for language.

**Method:**

Using a cross‐sectional design, language laterality was measured during an animation description task using functional transcranial Doppler ultrasonography. Data were available for 75 children with an SCT (47,XXX females [*n*=26], 47,XXY males [*n*=25], 47,XYY males [*n*=24]; mean age 11y 4mo [SD 3y 10mo]) and 132 comparison children with typical karyotypes (69 males, 63 females; mean age 9y 1mo [SD 1y 7mo]).

**Results:**

Lateralization for language did not differ between the SCT and comparison groups, either in mean laterality index or relative frequency of each laterality category. There were no differences when splitting the group with an SCT by trisomy. Handedness showed no group effects.

**Interpretation:**

Our data provide no evidence for disrupted lateralization for language in SCTs. The brain basis of the cognitive phenotype in SCTs is unlikely to be a failure of the left hemisphere to specialize for language, as previously suggested.

**What this paper adds:**

- Children with a sex chromosome trisomy (SCT) have typically lateralized language.
- This disproves theories linking language problems to hemispheric specialization in SCTs.

## What this paper adds

- Children with a sex chromosome trisomy (SCT) have typically lateralized language.
- This disproves theories linking language problems to hemispheric specialization in SCTs.

This article is commented on by van Rijn on page https://doi.org/10.1111/dmcn.13983 of this issue.

This article's abstract has been translated into Spanish and Portuguese.

Follow the links from the http://onlinelibrary.wiley.com/doi/10.1111/dmcn.13929/abstract to view the translations.

## Resumen

**Objetivo:**

Probar la hipótesis de que las trisomías cromosómicas sexuales (TCS) se asocian con una lateralización izquierda reducida para el lenguaje.

**Metodo:**

Utilizando un diseño de corte transversal, se midió la lateralidad del lenguaje durante una tarea de descripción de una animación mediante ecografía Doppler transcraneal funcional. Los datos fueron de 75 niños con TCS (47 mujeres XXX [n = 26], 47 varones XXY [n = 25], 47 varones XYY [n = 24], edad media 11 años y 4 meses [DS = 3 y 10 meses]) y 132 niños con cariotipos típicos para comparación (69 varones, 63 mujeres, edad media 9 años y 1 mes [DS = 1 año y 7meses]).

**Resultados:**

La lateralización del lenguaje no difirió entre el grupo con TCS y los grupos de comparación, ni en el índice de lateralidad media ni en la frecuencia relativa de cada categoría de lateralidad. No hubo diferencias al dividir el grupo TCS por trisomía. El uso preferido de una de las manos no mostró efectos de grupo.

**Interpretacion:**

Nuestros datos no proporcionan evidencia de lateralización interrumpida para el lenguaje en las TCS. Es poco probable que la base cerebral del fenotipo cognitivo en las TCS sea una falla del hemisferio izquierdo para especializarse en el lenguaje, como se sugirió anteriormente.

## Resumo

**Objetivo:**

Testar a hipótese de que as trissomias de cromossomos sexuais (TCS) são associadas com reduzida lateralização para a linguagem.

**Método:**

Usando um desenho transversal, a lateralidade da linguagem foi mensurada durante uma tarefa de descrição de uma animação usando ultrassonografia transcraniana de Doppler. Os dados estavam disponíveis para 75 crianças com TCS (47,XXX sexo feminino [*n*=26], 47,XXY sexo masculino [*n*=25], 47,XYY sexo masculino [*n*=24]; media de idade 11a4m [DP=3a 10m]) e 132 crianças de comparação com cariótipos típicos (69 do sexo masculino, 63 do sexo feminino; média de idade 9a1m [DP=1a 7m]).

**Resultados:**

A lateralização para linguagem não diferiu entre os grupos TCS e comparação, nem para a média do índice de lateralidade e nem para a frequência relativa de cada categoria de lateralidade. Não houve diferença quando o grupo TCS foi dividido por tipo de trimossia. A preferência manual não mostrou efeito de grupo.

**Interpretação:**

Nossos dados não fornecem evidência de alteração da lateralização para linguagem em TCSs. A base cerebral do fenótipo cognitivo provavelmente não é uma falha do hemisfério esquerdo em se especializar para a linguagem, como previamente sugerido.

**Abbreviations**
fTCD: Functional transcranial Doppler ultrasound
MCA: Middle cerebral artery
SCT: Sex chromosome trisomy

Sex chromosome trisomies (SCTs) are common genetic anomalies where an individual has an extra X or Y chromosome alongside the typical 46 chromosomes: males with an SCT are 47,XXY or 47,XYY, and females are 47,XXX. The risk of neurodevelopmental problems, especially language impairments, is increased among people with an SCT, as noted by a systematic review.^1^ Average Verbal IQ is around 1 standard deviation (SD) lower than in 46,XX or 46,XY karyotypes, and speech therapy is often required for early language difficulties. In males with an extra X or Y, non‐verbal ability is relatively unimpaired, whereas it is depressed in females. With this pattern of greater verbal than non‐verbal difficulties, SCTs may offer insights into developmental language disorder, in particular through understanding the neurobiological mechanism that mediates between the genetic anomaly and the language problems. The leading hypothesis is that an extra sex chromosome affects the dosage of genes involved in the typical asymmetric development of the brain,^2^, ^3^ which may disrupt the usual left‐sided bias for language function. A right‐sided or bilateral pattern may develop instead, possibly predisposing the individual to language disorder.

The existing evidence for atypical lateralization in individuals with an SCT is suggestive. We are aware of two studies that examined functional measures of activity in cerebral cortex, and found a reduced left bias at the group level.^4^, ^5^ A single‐photon emission computed tomography resting‐state study showed more bilateral perfusion of parietal and temporal regions in 47,XXY males (*n*=9) than in controls, who showed a left bias.^4^ In a functional magnetic resonance imaging study, mean laterality indices averaging activation across three language tasks indicated a reduced left bias across language‐relevant regions in 47,XXY individuals (*n*=15) versus control males (*n*=14).^5^ However, the data set contained an outlier: one 47,XXY participant was strongly right lateralized, whereas all other participants were left lateralized. With small samples, inclusion of the occasional person with reversed lateralization can have undue influence on the results. A further paper reported preliminary functional magnetic resonance imaging analysis of an 47,XXY sample (*n*=8) showing reduced left‐sided activation during a language task.^6^

There are mixed findings regarding structural asymmetries. Reduced left temporal lobe volume has been found in two samples of 47,XXY individuals (one of 15 individuals, although only after excluding left‐handers,^7^ and another of 10 individuals).^8^ However, neither study conducted direct between‐hemisphere comparisons. Two studies reported no differences in asymmetry, despite reduced overall brain volume in those with an extra X chromosome, in a sample including each SCT (*n*=34)^9^ and a 47,XXY sample (*n*=65).^10^ DeLisi et al. found similar results, with bilateral reductions in frontal and temporal volume but typical laterality in 47,XXY individuals (*n*=11), although three of four locations with reduced white matter integrity were left‐sided (left internal capsule, left arcuate, and bilateral anterior cingulate).^11^ Cerebral torque (the typical pattern of greater anterior volume in the right hemisphere and greater posterior volume in the left) has been examined in 47,XXY individuals (*n*=10) but did not differ significantly from controls.^12^ However, this study found reduced rightward asymmetry in white matter connecting the anterior commissure to the frontal lobe and a non‐significant trend (*p*=0.056) for reduced frontal asymmetry in the 47,XXY group. Reduced rightward asymmetry in the medial occipital lobe and superior temporal gyrus has been found in 47,XXY males versus control females (but not females).^13^

Although most neuroimaging work has focused on 47,XXY groups, a few studies have considered other SCTs. Right hemispheric regional differences have been found in 47,XYY males (*n*=10), even when controlling for greater overall volume in this group.^14^ The right occipital lobe showed increased grey matter volume, whereas the insula, inferior frontal gyrus, and superior temporal cortex volumes were decreased. Note, however, that direct comparisons between the hemispheres were not reported. A group of 47,XXX females (*n*=35) had decreased overall volume and regional reductions in frontal and temporal cortices, although laterality was not significantly different to controls.^15^ A final study examined whether a supernumerary sex chromosome affected cortical thickness in five sex chromosome aneuploidies (*n*=137), but found relatively typical torque, with only small foci of difference.^16^

While neuroanatomical differences have been found in individuals with an SCT, structural asymmetries seem unaffected. By contrast, functional neuroimaging studies, as well as behavioural studies using dichotic listening,^17^, ^18^ provide some evidence of a reduced bias for left‐sided language processing in 47,XXY individuals. This suggests that failure to establish a specialized language network in the left hemisphere may account for the verbal impairments of individuals with an extra sex chromosome.^2^, ^12^ However, evidence is limited to small exploratory studies, and calls for confirmation in a large sample, especially as the relationship between atypical laterality and developmental language disorder failed to replicate in a large sample of children with language problems without a known genetic aetiology.^19^ Even given this null result, it is possible that laterality is specifically affected in SCTs, perhaps through the effects of increased gene dosage on brain development. Therefore, in this study, we hypothesized that children with an SCT would show a reduced bias for left‐sided blood flow during a language task, compared to children with a typical karyotype. We also tested for reduced right‐handedness in the group with an SCT, given the link between manual and language laterality.

## Methods

### Participants

Using a cross‐sectional design, we compared language laterality and handedness in children with an SCT aged between 6 years and 15 years 11 months (*n*=75) with a group of twin children aged between 6 years and 11 years 11 months (*n*=132). Our previous paper gives details of the twin sample, which did not vary in laterality from single‐born children.^19^ As independence of observations cannot be assumed among twins, only one twin from each pair was included in the present analysis: the child randomly labelled ‘twin 1’.

**Figure 1:**
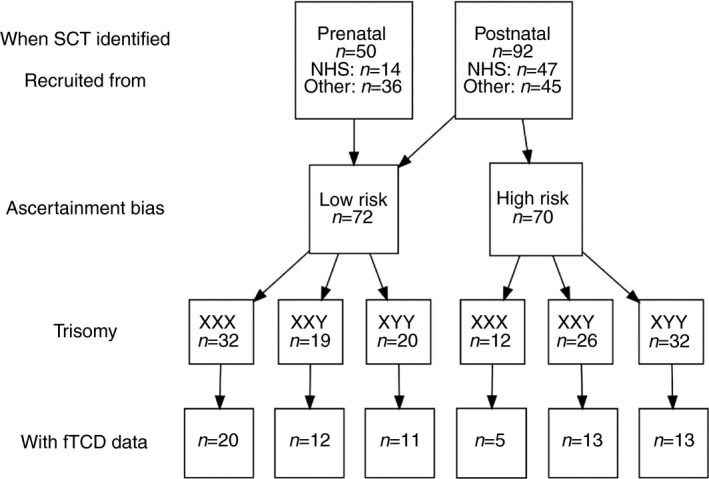
Chart showing the flow of children with a sex chromosome trisomy (SCT) through the study. The subgroup of children marked as having a high risk of bias were diagnosed postnatally in a medical evaluation after neurodevelopmental or behavioural concerns. Functional transcranial Doppler ultrasound (fTCD) data were not available for all of the children for the following reasons: inability to establish ultrasound signal (*n*=18); poor signal (*n*=9); child refusal (*n*=19); child non‐compliance (*n*=2); task too difficult for the child (*n*=8); insufficient time during testing (*n*=7); error with the recording (*n*=5). Note that one child (a 47,XXX female) is not included, as her early history was not known. NHS, National Health Service.

Out of 143 children with an SCT recruited into the study, we collected useable functional transcranial Doppler ultrasound (fTCD) data from 75 (Fig. 1). Recruitment was via National Health Service Clinical Genetics centres, two support groups (Unique: the Rare Chromosome Support Group; and the Klinefelter Syndrome Association) and self‐referral through social media. An inclusion criterion was that children were aware of their trisomy status. During the initial telephone interviews, caregivers were asked how their child was diagnosed, in particular whether this followed postnatal testing motivated by neurodevelopmental/behavioural problems. The phenotype of such children may be more severe, potentially biasing the sample, and so we grouped them prospectively as a high‐risk‐of‐bias subgroup. All other children formed the low‐risk‐of‐bias subgroup. This analysis strategy follows previous research in this population.^20^

### Measures

Details of language and psychopathology assessments are reported elsewhere.

#### Handedness assessments

We used an adapted version of the Edinburgh Handedness Inventory to measure hand preference, replacing one item (striking a match) with a more child‐friendly one (dealing cards).^21^ The child was asked to demonstrate how they would perform 10 actions. For each action, exclusive right‐hand use scored 1 point, left hand use 0 points, and both hands 0.5. Totals out of 10 were converted to indices between –100 (extreme left‐handedness) and 100 (extreme right‐handedness).

The Quantification of Hand Preference task measured strength of hand preference.^22^ This assesses tendency to reach across the midline with the preferred hand. Twenty‐one picture cards were arranged in front of the child, three stacked in seven positions at 30‐degree intervals. The child placed named cards into a central box one at a time in the same quasi‐random order used for all participants. The child was not told that the task assessed handedness. Each right‐handed reach scored 1 point and use of both hands scored 0.5 points. Total scores were therefore out of 21.

In addition to the quantitative handedness indices provided by these two measures, we also assigned each child a categoric handedness based on each of these measures. Scores higher than 0 indicated right‐handedness by the adapted Edinburgh Handedness Inventory, and scores higher than 10 indicated right‐handedness by the Quantification of Hand Preference.

#### Language laterality assessment

Language laterality was assessed using fTCD (see our related paper for a full description of the procedure and data analysis).^19^ Briefly, ultrasound probes were positioned to detect blood flow in the left and right middle cerebral arteries (MCAs). The child performed an animation description task comprising a maximum of 30 trials. Each trial involved watching a 12‐second cartoon, before describing it during a 10‐second talk phase. Trials were excluded where the child spoke during a silent period, or said nothing during the talk phase.

Analysis of the fTCD data involved comparing blood flow during the period of interest where the child speaks with the baseline period spent watching the cartoon. The period of interest was 10 seconds in length and began 4 seconds into the talk phase to allow for task‐related blood flow changes to have happened. After initial data processing, including exclusion of trials with poor signal, a laterality index was calculated for each child. Firstly, the velocity in both the left and right MCAs was averaged across all trials for each time point; this gave mean curves for velocity in each artery during the time course of a trial based on all the data collected. Secondly, we identified the maximum difference between these two curves during the period of interest, and a 2‐second window was constructed around this maximum point. Velocity was averaged throughout this window for both MCAs, and the laterality index was given by subtracting the right average from the left. Children with laterality indices based on less than 12 trials were excluded from analysis, since these laterality indices are unlikely to be reliable. The threshold was placed at 12 based on previous experience that this represents an optimal trade‐off between losing participants because of too few trials and maintaining low measurement error.^23^

Participants were also assigned a laterality category. Ninety‐five per cent confidence intervals (CIs) were calculated around the laterality index. If the CIs did not cross zero, laterality was classified as left or right depending on direction, and bilateral where the CIs crossed zero. Note, however, that laterality may be identified as bilateral if data are merely noisy (95% CIs were calculated using the standard error [SE] derived from trial‐by‐trial laterality indices computed for every valid trial during the same 2s window as the overall laterality index).

Laterality indices were derived separately from odd and even trials to allow computation of split half reliability, and the mean number of words spoken by the child during valid trials was recorded.

#### Non‐verbal ability and language status

Non‐verbal ability was estimated using the two non‐verbal subtests of the Wechsler Abbreviated Scale of Intelligence: Block Design and Matrices.^24^ Scores were converted to Performance IQ.

A battery of 13 tests assessed language abilities.^19^ In determining language status, performance at least 1 SD below the normative mean on two or more language tests indicated language problems. Otherwise, the child had typical language.

### Procedure

Ethical approval was obtained in 2011 from the Berkshire NHS Research Ethics Committee (reference 11/SC/0096). Data were collected between August 2011 and October 2016 from families throughout the UK. Families were first interviewed by telephone, and if inclusion criteria were met, an assessment was arranged at home or school. Written consent was obtained from a parent/caregiver, and children signed a simplified assent form. Eight research assistants and the senior author conducted assessments, scheduled in a single session lasting 2 to 3 hours.

### Statistical analysis

See Appendix S1 (online supporting information) for details of (1) software used in analysis and (2) data storage and availability.

**Figure 2:**
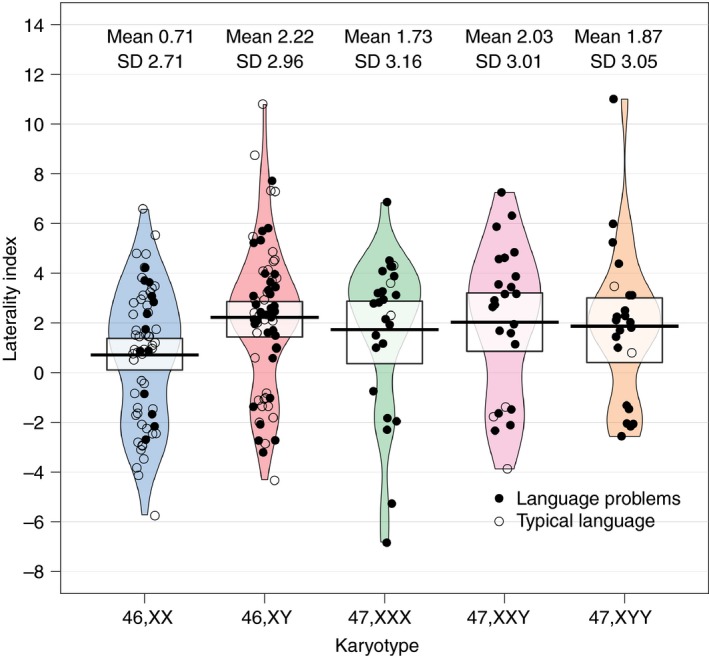
Pirate plot showing distributions of laterality indices for the children with sex chromosome trisomy and comparison children, split by karyotype. The 46,XX and 46,XY children constitute the comparison group. All data are shown with smoothed densities indicating the distributions in each subgroup. The central tendency is the mean and the intervals are Bayesian 95% highest density intervals. [Colour figure can be viewed at http://wileyonlinelibrary.com].

For our main analysis, we tested the hypothesis that the group with an SCT would show a reduced bias for left‐lateralized blood flow during a language task. Response variables were quantitative laterality index and laterality category. Multiple linear regression was used to test for a group effect (SCT or comparison group) on laterality index, controlling for age and sex. Pirate plots were used to visualize the individual variability in laterality indices found for the children in our sample based on their karyotype and language status (see Fig. 2): these provide an economical format for showing individual data, as well as distributional features in a single plot. A multinomial logistic regression tested whether group (SCT or comparison) predicted laterality category, controlling for age and sex. This model estimated two logit equations, each comparing relative frequency of left‐lateralized language to one atypical laterality (bilateral and right), and assigned predicted log‐odds to each predictor. We planned to test whether any evidence for atypical laterality in the group with an SCT was not influenced by risk of bias, by testing for differences in prospectively defined high‐ and low‐risk‐of‐bias subgroups. We also examined whether handedness was atypical in the children with an SCT. As explained in our previous paper,^19^ the two quantitative handedness measures are best modelled using inflated beta regression. We ran one model per measure; the logit function of the handedness measure was response variable and group (SCT or comparison), age, and sex were predictors.

## Results

Table 1 provides a summary of the descriptive statistics.

**Table 1:** Summary statistics

	SCT group	Comparison group
Sample characteristics		
Children (*n*)	75^a^	132^b^
Age (y:mo)	11:4 (3:10)	9:1 (1:7)
Male sex	49 (65)	69 (52.3)
Performance IQ	93.16 (16.17)	103.15 (13.57)
Language status^c^	67 (89)	48 (36.4)
fTCD language laterality		
Total trials completed	24.75 (5.25)	26.83 (3.22)
Total words produced per trial	17.70 (6.19)	19.90 (5.12)
Laterality index	1.87 (3.04)	1.50 (2.93)
Left language (total children)	47 (63)	85 (64.4)
Bilateral language (total children)	14 (19)	21 (15.9)
Right language (total children)	14 (19)	26 (19.7)
Handedness		
EHI	63.07 (58.01)	62.50 (61.04)
Total children right‐handed by EHI	64 (85.3)	110 (83.3)
QHP	13.19 (6.96)	14.18 (7.38)
Total children right‐handed by QHP	51 (68)	95 (72)

All continuous variables are reported as mean (SD) and categoric variables are reported as *n* (%). ^a^Performance IQ only available for 68/75 children with a sex chromosome trisomy (SCT). ^b^Performance IQ only available for 131/132 comparison children. ^c^Number with language problems. fTCD, functional transcranial Doppler ultrasound; EHI, Edinburgh Handedness Inventory; QHP, Quantified Hand Preference.

### Preliminary analysis of fTCD data

We considered whether there were any group differences that may affect interpretation of any laterality findings. Welch's *t*‐test revealed that fTCD data for the children with an SCT included fewer valid trials (*t* [106.19]=–3.12 [*p*=0.002], Cohen's *d*=0.45). The average child with an SCT also produced slightly fewer words per valid trial (*t* [126.89]=–2.59 [*p*=0.011], Cohen's *d*=0.38). Next we checked whether task performance (operationalized as number of words produced per trial) correlated with laterality index; it did not (*r*=0.002, *p*=0.973). Mean number of words produced by left‐lateralized children was 19.38 (SD 5.63) versus 17.71 (SD 5.81) for those with bilateral language and 19.49 (SD 5.31) for the right‐lateralized children.

Finally, split half‐reliability of the laterality indices was high in both groups (SCT *r*=0.87, comparison *r*=0.84), indicating that laterality indices represented stable cerebrovascular responses.

### Hypothesis testing

**Figure 3:**
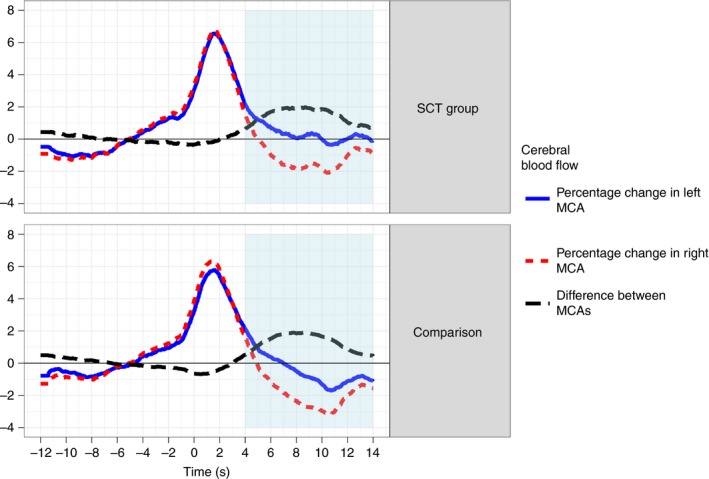
Plot showing grand average curves in the two groups (sex chromosome trisomy [SCT] and comparison) for blood flow velocity in the left and right middle cerebral arteries (MCAs) during the time course of a trial. The curves for the two MCAs represent percentage change in blood flow velocity based on the average across the baseline. The third curve represents the absolute difference in percentage change in velocity between the left and the right. For reference, the child speaks between 0 seconds and 10 seconds, and the period of interest (during which the laterality index is identified) runs from 4 to 14 seconds. Immediately before they start speaking, the child is watching a 12‐second cartoon, which is taken to be the baseline period. [Colour figure can be viewed at http://wileyonlinelibrary.com].

See Figure 2 for a pirate plot showing laterality index distributions, and Figure 3 for a plot showing the time course of MCA blood flow.

#### Language laterality

We tested our main hypothesis that mean laterality index would be reduced in the children with an SCT, when controlling for age and sex, using multiple regression. The group factor was not significant (*β*=0.07, SE=0.16 [*p*=0.663]), indicating, contrary to the hypothesis, that both groups showed no difference in the usual left‐sided bias for language. Age was not significant (*β*=0.01, SE=0.07 [*p*=0.860]), although sex was (*β*=–0.36, SE=0.14 [*p*=0.011]). The effect of sex has already been reported for the comparison group,^19^ so we ran a post hoc *t*‐test to test for a sex difference in the group with an SCT, which found no difference (*t* [48.78]=0.29, *p*=0.773).

Visual inspection of the pirate plot indicates no differences in laterality index by trisomy (47,XXX; 47,XXY; or 47,XYY). Likewise, there were no laterality differences when analysing the children with an SCT by possible risk of bias based on circumstances of diagnosis. Mean laterality index of the high‐risk‐of‐bias subgroup was 2.32 (SD 3.31) versus 1.72 (SD 2.66) for the low‐risk‐of‐bias children (*t* [55.83]=0.83, *p*=0.409).

We also checked for group differences in proportions of children with each laterality category. A multinomial logistic regression tested whether the odds of being atypically lateralized differed by group, when controlling for age and sex. Comparing bilateral with left laterality, the predicted odds for all factors were non‐significant: group 1.58 (95% CI 0.70–3.58; *p*=0.268), age 0.74 (95% CI 0.49–1.11; *p*=0.144), and sex 1.83 (95% CI 0.85–3.94; *p*=0.124). Comparing right against left laterality, all factors were, again, non‐significant: group 0.79 (95% CI 0.33–1.91; *p*=0.597), age 1.30 (95% CI 0.87–1.95; *p*=0.124), and sex 1.52 (95% CI 0.74–3.13; *p*=0.124). This analysis confirms that language laterality was not unusual in the children with an SCT.

#### Handedness

Inflated beta regressions indicated that there were no significant effects of group, age, or sex on either of the two handedness measures. The predicted odds that a child with an SCT in relation to a comparison child was fully right‐handed rather than left‐handed on the adapted Edinburgh Handedness Inventory was 1.12 (95% CI 0.74–1.71; *p*=0.597). The predicted odds that a child with an SCT in relation to a comparison child was fully right‐handed rather than left‐handed on the Quantification of Hand Preference was 0.83 (95% CI 0.57–1.22; *p*=0.341).

## Discussion

The present study found no differences in cerebral lateralization for language in 75 children with an SCT. Previous research has offered weak support for structural differences in brain asymmetry in individuals with an SCT, although two small functional studies did report atypical lateralization in 47,XXY individuals.^4^, ^5^ One was a resting state study,^4^ and, conceivably, differences in participant behaviour in the scanner contributed to the reduced functional asymmetry in the clinical group. The other functional neuroimaging study may have been distorted by an outlier,^5^ as discussed in the introduction. The present results question the leading theoretical accounts of the brain basis of the language phenotype in SCTs, and we outline points of contention below.

Rezaie et al.^12^ summarized the view that the SCT phenotype represents a left hemisphere dysfunction, as impairment in a typically left‐lateralized function, language, is a hallmark feature. These researchers contrasted SCTs with Turner syndrome (where a female has one X chromosome rather than two), which is characterized by impaired visuospatial cognition, a typically right‐lateralized function. This link between sex chromosome aneuploidies and lateralized cognitive functions has been taken as evidence that genes on the sex chromosomes influence lateralization.^3^ A high dosage of such genes, when an individual has an extra sex chromosome, may disrupt lateralization of left hemisphere function (whereas, for some unspecified reason, low dosage in Turner syndrome affects the right hemisphere). However, the lack of effects of an extra sex chromosome on laterality in the present data provides no evidence for these hypothetical laterality genes.

An alternative theory is based on the hypothesis that the left hemisphere develops more rapidly, and has an inhibitory effect on the right, in most people.^17^ This typical trajectory may be disrupted in individuals with an SCT owing to a loss of the left‐on‐right inhibitory mechanism, such that the right hemisphere assumes some language function, contributing to reduced processing efficiency. Inefficient organization of language function across the cerebral hemispheres has been theorized elsewhere as a factor in developmental language disorder.^25^ However, this theory was not supported by analysis of a large sample of typically developing and language‐impaired children (*n*=267), who showed no laterality differences.^19^ As for the present study, 90% of the children with an SCT had language difficulties diagnosable as developmental language disorder, but there was no evidence of atypical laterality. Hemispheric specialization for language was usually left‐lateralized.

Theoretical accounts also implicate atypical cerebral laterality in the elevated psychiatric risk associated with SCTs. The most documented risk is for autism.^20^ With relevance to the present study, theories have linked the communication difficulties in autism with atypical lateralization and left hemisphere dysfunction.^26^, ^27^ In addition, the 47,XXX and 47,XXY karyotypes have been explored as genetic models for schizophrenia,^5^ although an increased risk for schizophrenia is not well established.^28^ Nonetheless, the relationship between atypical lateralization and schizophrenia,^29^ and the possibility that genes on the sex chromosomes influence lateralization and psychosis,^3^ make a putative link between SCTs, schizophrenia, and lateralization of substantial psychiatric interest. However, our null findings speak against the view that atypical laterality may be an endophenotype representing increased psychiatric risk in SCTs.

A limitation in the present study is that fTCD may fail to detect fine‐grained laterality differences, because it measures blood flow changes in the MCA, which covers a wide territory. The possibility remains that small regional differences in lateralization may go undetected by this method. It should also be noted that changes in blood flow velocity cannot be assumed to vary proportionally with changes in blood flow volume, given that arterial diameter may change as well,^30^ and so velocity may not directly reflect metabolic demand.^31^ However, this does not alter the observation that fTCD is sensitive to the left‐sided laterality bias expected for language, with greater blood flow velocity measured on the left compared with the right. We can therefore be confident that fTCD is detecting task‐related vascular reactivity.

## Conclusion

In this relatively large study of children with SCTs, we found no evidence for atypical lateralization for language. The proportion of children showing left, bilateral, and right‐lateralized language was identical with a comparison group, and mean laterality indices did not vary by karyotype. In a previous study, we showed that lateralization for language was not disrupted in children with disordered language.^19^ The present study develops upon that finding by indicating that a specific genetic disorder linked to language problems also does not disrupt typical lateralization. This result disproves a leading hypothesis that atypical lateralization for language is the neurobiological basis for the cognitive phenotype in SCTs.

## Supporting information


**Appendix S1:** Data analysis.
