# Supplementary material for: Sex chromosome trisomies are not associated with atypical lateralization for language
Source: Dev Med Child Neurol. 2018 Jun 10;60(11):1132–9. doi: 10.1111/dmcn.13929 (PMC6220794; doi:10.1111/dmcn.13929)
Supplement: Supplementary file 1 — Appendix S1: Data analysis. [file DMCN-60-1132-s001.docx]

**Appendix**

Study data were analysed using R software^1^, with the main database managed using REDCap, hosted at the University of Oxford^2^. Original data are available on *Open Science Framework* at <https://osf.io/2w6u5/>.

The multinomial logistic regressions were run in R using the nnet package^3^. The inflated beta regressions were implemented with the GAMLSS package^4^. The flow chart was produced using the DiagrammeR package^5^, the plot showing changes in MCA blood flow with ggplot2^6^, and the pirate plot with yarrr^7^. The R packages tidyverse^8^, stringr^9^, psych^10^, and xlsx^11^ were also used in analysis.

**References**

1. R Core Team. R: A language and environment for statistical computing. 2016. Available from: <https://www.r-project.org/>

2. Harris PA, Taylor R, Thielke R, et al. Research electronic data capture (REDCap): A metadata-driven methodology and workflow process for providing translational research informatics support. *Journal of biomedical informatics* 2009; 42: 377–381.

3. Venables WN, Ripley BD. *Modern Applied Statistics with S*. 4th Edn. New York, NY: Springer, 2002.

4. Stasinopoulos DM, Rigby RA. Generalized additive models for location scale and shape (GAMLSS) in R. *Journal of Statistical Software* 2007; 23: 1–46.

5. Sveidqvist K, Bostock M, Pettitt C, et al. DiagrammeR: Create Graph Diagrams and Flowcharts Using R. R package version 0.9.2; 2017. Available from: <https://cran.r-project.org/package=DiagrammeR>

6. Wickham H. *ggplot2: Elegant Graphics for Data Analysis*. Springer-Verlag New York, 2009.

7. Phillips N. yarrr: A Companion to the e-Book ‘YaRrr!: The Pirate’s Guide to R’. R package version 0.1.5; 2017. Available from: <https://cran.r-project.org/package=yarrr>

8. Wickham H. tidyverse: Easily Install and Load ’Tidyverse’ Packages. R package version 1.1.1; 2017. Available from: <https://cran.r-project.org/package=tidyverse>

9. Wickham H. stringr: Simple, Consistent Wrappers for Common String Operations. R package version 1.2.0; 2017. Available from: <https://cran.r-project.org/package=stringr>

10. Revelle W. psych: Procedures for Psychological, Psychometric, and Personality Research. R package version 1.7.8; 2017. Available from: <https://cran.r-project.org/package=psych>

11. Dragulescu AA. xlsx: Read, write, format Excel 2007 and Excel 97/2000/XP/2003 files. R package version 0.5.7; 2014. Available from: <https://cran.r-project.org/package=xlsx>
